# Individual differences in vicarious pain as a shift in the self–other boundary

**DOI:** 10.1162/imag_a_00422

**Published:** 2025-01-21

**Authors:** Mengze Li, Chris Racey, Samira Bouyagoub, Hugo D. Critchley, Jamie Ward

**Affiliations:** School of Psychology, University of Sussex, Brighton, United Kingdom; Department of Military Medical Psychology, Air Force Medical University, Xi’an, China; Brighton and Sussex Medical School, University of Sussex, Brighton, United Kingdom

**Keywords:** vicarious pain, empathy, emotion contagion, fMRI, multi-voxel pattern analysis

## Abstract

There is inconsistent evidence concerning whether physical pain and vicarious pain share neural resources. This may reflect different methodological approaches (e.g., univariate vs. multivariate fMRI analyses) and/or participant characteristics. Here we contrast people who report experiencing pain when seeing others in pain (vicarious pain responders) with non-responders (who do not report pain). Cues indicated the level and location of an electrical shock delivered to the participant (self) or experimenter (other), with behavioural ratings and neural responses (fMRI) obtained. Non-responders tend to rate their own pain as worse than others given identical cues, whereas responders show greater similarity between self and other ratings. Univariate neuroimaging analyses showed activity in regions of the pain matrix such as insula, mid-cingulate, and somatosensory cortices contrasting physical versus vicarious pain, and when regressing the level of self-pain. But these analyses did not differ by group. Multivariate analyses, by contrast, revealed several group differences. The ability to classify self versus other was less accurate in the vicarious pain responders (in the same regions implicated in the univariate analyses of physical pain). In conclusion, the degree of shared neural responses to physical and vicarious pain is increased in vicarious pain responders consistent with the notion of differences in the self–other boundary.

## 1 Introduction

To what extent is seeing someone else in pain like the experience of being in pain? For many years, the consensus view amongst the neuroscience community was that there was an intimate connection between these two processes—which we refer to as vicarious pain and physical pain, respectively. This evidence was primarily motivated by neuroimaging studies (using fMRI) showing that seeing someone else in pain and experiencing pain on one’s own body activated the same neural regions, including the mid-cingulate cortex and anterior insula (Lamm et al., 2011). Converging evidence comes from experimental manipulations; for instance, certain drugs (opioid antagonists) exert an effect on neural responses to both physical pain and vicarious pain (Ruetgen et al., 2015). This research suggests the existence of “shared representations” for pain that bridge between self and other, also called mirror systems (Keysers et al., 2010). However, this consensus view has received a number of significant challenges in recent times. Firstly, there is a suggestion that these shared neural responses are not really representing pain, but instead represent more general constructs relating to pain such as arousal or negative affect (Ferris et al., 2019;Iannetti et al., 2013). Secondly, others have gone further by arguing that shared representations for pain do not exist at all. A brain region may appear to be activated by both vicarious pain and physical pain when looking for broad regions of overlap (so-called univariate analyses). But this could reflect distinct and independent neural populations within this region that are only observed when looking at finer spatial patterns of activity (so-called multivariate analyses) (Krishnan et al., 2016). The latter study, byKrishnan et al. (2016), provided the main motivation for the present research. Specifically, we test the hypothesis that shared representations between physical and vicarious pain are more likely to be found in certain people—those who have reportable pain-like experiences when seeing others in pain.

Multivariate fMRI techniques use machine learning algorithms on fine-grained patterns of brain activity (on a voxel scale) in order to predict specific outcomes, such as the level of pain. Using this approach,Krishnan et al. (2016)identified a pattern of brain activity that predicted intensity ratings for physical pain (which they termed the “neurologic pain signature”) and a pattern of brain activity that predicted intensity ratings for vicarious pain when rating imagined pain levels of other people in response to images (e.g., of a trapped finger). Crucially, these patterns were not predictive of each other (e.g., the pattern for physical pain was not predictive of vicarious pain ratings) and loaded on to different regions. The “neurologic pain signature” was derived from regions such as insula and somatosensory cortex (involved in body sensations), whereas the “vicarious pain signature” loaded on to regions involved in mentalizing (including right temporoparietal junction). When univariate activations linked to physical pain and vicarious pain are simply overlayed (rather than applying classifiers), then many of these regions overlap. But, according to the authors, they are an artefact of smoothing and averaging across voxels (as opposed to being driven by shared representations between physical and vicarious pain). Another possibility is that shared representations between physical and vicarious pain do exist but are limited to a minority of the population and, hence, tend to be averaged out at group-level findings.

As many as one quarter of the population report that, when seeing others in pain, they have a subjectively real and consciously experienced feeling of pain themselves (Grice-Jackson et al., 2017a). We refer to these people as vicarious pain responders and, using a measure that we developed (the vicarious pain questionnaire, VPQ), we show that they can be split into two sub-types. One group, termed sensory/localized (S/L) responders, reports localized feelings of pain (e.g., seeing someone trap their finger and feeling it on their own finger) and uses sensory-based pain adjectives. The other group, termed affective/general (A/G) responders, report non-localized pain and use emotion-based pain adjectives. The remainder of the population (around three-quarters) report no subjective pain experiences and can be regarded as either having an unconscious vicarious pain response or, perhaps, a vicarious pain response that does not depend at all on neural substrates involved in physical pain perception. These individual differences in vicarious pain experience within the general population (measured with the VPQ) map on to structural and functional brain differences (Grice-Jackson et al., 2017a), cognitive differences relating to body perception (Botan, Fan, et al., 2018), and social–emotional behavior (Botan, Bowling, et al., 2018). Specifically, on questionnaire measures of empathy, responders identified via the VPQ report higher level of emotion contagion and emotional reactivity but do not necessarily differ in other ways such as deliberate perspective taking (Botan, Bowling, et al., 2018).

Vicarious pain, and other forms of shared experience (or emotion contagion), can be conceptualized as a reduction in the boundary between self and other. This can arise from a set of mechanisms that infer the origins of sensory signals (e.g., to determine whether an observed body is likely to be one’s own) and can actively control which information to select (e.g., to attend to one’s self without interference from another, and vice versa) (Bird & Viding, 2014). Individual differences in these mechanisms could give rise to experiencing the pain of others, in addition to this wider profile of differences, i.e., beyond pain (Ward & Banissy, 2015). Pain is a multi-faceted experience, sometimes divided into sensory and affective components, and vicarious pain may differentially load on to some components more than others. This could vary across contexts depending on the extent to which different kinds of information is present (e.g., seeing an injection to a precise bodily location might bias toward sharing of sensory pain components;Lamm et al., 2011). Different vicarious pain components might also vary as traits across individuals. Intuitively, sensory/localized (S/L) responders may have greater sharing of somatotopically organized representations of pain, whereas affective/general (A/G) responders may preferentially share other aspects of pain (e.g., interoceptive feeling states). In support of this, S/L responders show differences in an EEG signal linked to sensorimotor regions (Grice-Jackson et al., 2017a), and A/G responders have differences in interoceptive accuracy (Botan et al., 2021).

The present study administered pain, in the form of a mild electric shock, in an fMRI scanner and, on different trials, presented vicarious pain. There are multiple ways of eliciting vicarious pain but they can be broadly divided into cue-based procedures (where pain is inferred) and picture-based procedures (where pain is directly observed) (Lamm et al., 2011). Both procedures have their pros and cons. Picture-based procedures have frequently made use of the stimulus set ofJackson et al. (2005), which consists of hands or feet in painful or neutral poses. The advantage of these is that they are more likely than cue-based procedures to activate somatosensory cortices (Lamm et al., 2011;Xiang et al., 2018). However, they are less suited for addressing shared representations because the same kind of physical pain cannot be administered experimentally. Cue-based procedures, such as those used bySinger et al. (2004), often use an arrow to denote which person (self or other) will receive a shock. In this approach, the type of pain is matched between self and other although the participant does not see pain as such (i.e., in the form of injury, or a bodily expression of pain). Cue-based paradigms also involve pain delivered in real time. The present study describes the results of a cue-based pain paradigm, noting that a separate picture-based paradigm (on the same participants) is described elsewhere (Li et al., 2024). We hypothesize that, in responders (both S/L and A/G), we expect to see a greater activity of the pain matrix in the vicarious pain conditions (using univariate analyses) and, moreover, that the multivariate pattern for physical pain will also predict vicarious pain (consistent with the notion of “shared representations”). There has been no previous attempt to directly compare vicarious pain and physical pain in these groups using fMRI. We would also predict differences between responders such that those with an S/L profile are more likely to engage sensory and somatotopically organized regions, such as primary somatosensory cortex, during vicarious pain.

## 2 Methods

Participants completed the study over three sessions: (1) the online VPQ questionnaire to determine their group status and select for the further sessions, (2) a behavioral session to establish pain thresholds and introduce them to the empathy for pain procedure, and (3) the neuroimaging session (seeFig. 1). The main task in the behavioral and neuroimaging session consisted of an empathy for pain procedure similar to that used by others (Singer et al., 2004) in which either the participant or another person (the experimenter) receives a shock following a cue. Visual cues indicate the part of body to be stimulated (hand or foot), the person being stimulated (self or other), and level of pain (high, medium, low). A second picture-based task was also included in the fMRI session in which participants passively watch standard pictures of hands and feet either in pain or no pain, and this is reported elsewhere (Li et al., 2024).

**Fig. 1.**
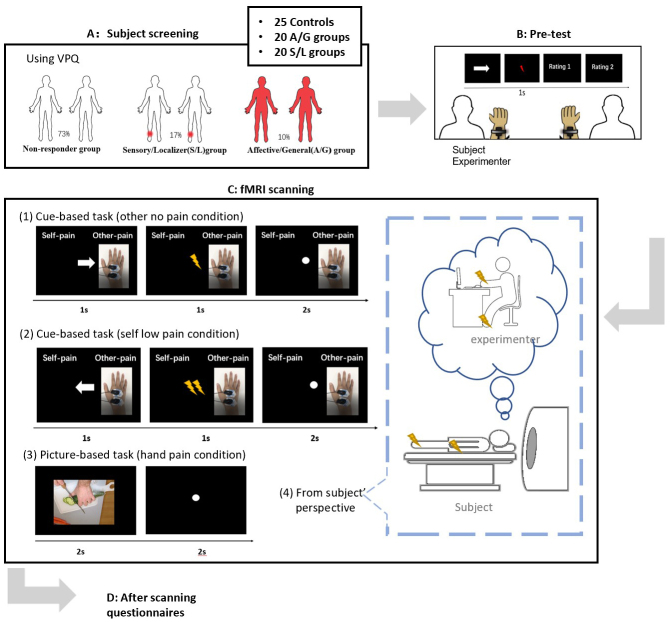
Schematic representation of the different sessions and the experimental design. (A) Subject screening is done online. (B) A version of the scanner task is done in a pre-test with participant seated side-by-side. (C) Examples of trials in the neuroimaging session. (D) Questionnaires are completed after scanning. (1) Example of trial design for other no pain condition in cue-based task. (2) Example of trial design for self low pain condition in cue-based task. (3) Example of trial design for hand pain condition in picture-based task. (4) The participant is told that the experimenter is sitting in the control room and her hand/foot is shocked exactly the same as the subject.

### 2.1 Participants

Participants were recruited from the student population of the University of Sussex, UK, who completed the VPQ for course credits. A clustering analysis of the scores classified participants into one of three groups—non-responder, S/L responder, and A/G responder (Ward & Li, 2022). From this, we recruited at least 20 participants from each group to the main study (with one subsequently excluded from the neuroimaging analysis). For the non-responders, we recruited 25 participants, and their mean age are 20.28 years (SD = 2.36, men:women = 8:17). For the A/G responders, we recruited 20 participants, and their mean age are 21.45 years (SD = 3.15, men:women = 4:16). For the S/L responders, we recruited 20 participants, and their mean age are 22.60 years (SD = 6.85, men:women = 5:15). Groups did not differ significantly in terms of either age or gender (ethnicity was not recorded). All participants have no history of psychiatric, neurological, or pain disorders and no current pain symptoms, and all of them had normal or corrected-to-normal visual acuity. All were right handed. In the vicarious pain conditions, the “shocks” were delivered to a woman (the first author), previously unknown to participants. We ran a sensitivity analysis to confirm that the smallest effect that we could detect in our least powered (between-subjects) test was a reasonable minimum effect size of interest. The sensitivity analysis showed that we can detect effects with a size of r^2^= 0.14 or larger, with an 80% probability.

All the subjects provided informed consent at the beginning of the study, and they were paid £20 for their participation in the in-person sessions. This study was reviewed and approved by the Brighton and Sussex Medical School (BSMS) Research Governance and Ethics Committee of the University of Sussex.

### 2.2 Materials and procedure

#### 2.2.1 Vicarious Pain Questionnaire (VPQ)

This was administered online to a large cohort of students (~500 over 2 years) and was used to screen participants and invite them to further research (including but not limited to the present study). The VPQ consists of a set of 16 video clips depicting real pain (injections, and minor sport injuries such as falling off a skateboard) (Grice-Jackson et al., 2017a). Participants are asked to report whether they experience any pain in their own body (giving a 0–16 score across all videos) and, as appropriate, follow-up questions asking whether the pain was localized or not (giving a localized minus generalized score), and a checklist of sensory and affective pain descriptors (giving a sensory minus affective score). These three variables have good test–retest reliability (Spearman’s rho of 0.629, 0.295, 0.550;Botan, Fan, et al., 2018) and are used to generate the three groups via a two-stage hierarchical followed by k-mean clustering process (Zhang et al., 1996). Novel participants can be classified according to their nearest z-scored distance to one of the three cluster centroids (Ward & Li, 2022). This avoids the need to rerun the clustering process each time the sample is updated (which would also lead to some instability in the group classification).

#### 2.2.2 Behavioural pre-imaging session

Firstly, all participants completed a pain calibration session to determine the electrical stimulation levels that they would receive in the following fMRI experiment and to introduce the task.

Electrical stimuli were delivered by a constant-current stimulator (DS7A, Digitimer Ltd., Welwyn Garden City, UK). The initial electrical stimulus was 1 mA, incrementing by 0.2 mA, and the subjects were asked to rate their pain on a scale of 0 to 10 (0 represents no sensation, 1 represents a touch sensation, 5 represents low pain, and 10 represent high but tolerable pain). The electrical current corresponding to the low- and high-pain sensations were then carried forward. This was repeated for both right hand and right foot (with the order counterbalanced across participants).

After the calibration, they would later complete a “self-pain and other pain task” similar to the cue-based task in the scanner. The participant and experimenter were seated next to each other (participant on the left) with electrodes attached to the hands in one block and feet in another. Electrodes were attached to the participant’s right hand or foot and the experimenter’s left hand or foot so that both were clearly visible next to each other (and so that the experimenter could use her right hand to control the study). One block stimulated hands, and the other feet.

An arrow cue indicated who would receive the pain and yellow lightning bolts (1, 2, or 3) indicated the approximate level of pain. When the subject was given the shock, they had to answer one question using 0–10 scale: “How intense was the pain for you?” When the researcher was given the shock, the subject had to answer two questions using the 0–10 scale: Q1 “How intense was the pain for the researcher?” and Q2 “How intense was the pain for you while you were observing?” There were 30 trials in each block with 3 stimulation levels used (corresponding to subjective values of 0, 5, and 10 on the 0–10 scale). The researcher would react to the shocks, verbally and non-verbally, in a similar way to the participant to make the paradigm realistic (noting that no shock was actually delivered to the experimenter).

#### 2.2.3 Neuroimaging session

This session was scheduled on a separate day from the behavioural session. In the scanner, visual stimuli were presented using MATLAB (version 2018a; The MathWorks, Inc., Natick, MA, USA) and Psychophysics Toolbox extensions version 3 (Brainard, 1997) on a screen viewed via a mirror mounted on the head coil.

Electrical shock stimuli were delivered using a dual channel STMEPM-MRI electrical stimulation system (BIOPAC Systems Inc, Goleta, CA, USA). Participants were invited to lie in the scanner and had electrodes attached to the back of their right hand and right foot, and the pain levels checked again. As the pain stimulators for pre-test and scanning were different models (one based on current [I], the other on voltage [V]), we calibrated the low and high pain levels for them using an independent sample of participants (N = 20). Specifically, the resistance (R) was estimated from the formula (V = I x R) to be ~860 k Ohm (hands) and ~1090 k Ohm (feet), enabling us to estimate voltage (in the scanner) from current (in the pre-test).

Skin conductance and heart rate data were collected using a BIOPAC MP160 system sampling at 1000 Hz. All skin conductance electrodes were placed on the left hand. We measured skin conductance with two EL509 EDA isotonic gel electrodes with 11 mm Ag/AgCl contact attached to the head of the index and middle fingers. All ECG electrodes were placed on the chest of the participant. We measured heart rate with EL101 gel and EL508 electrodes with 11 mm Ag/AgCl contact. (The psychophysiological measures are not reported here and will be analysed separately.)

The scanning session consisted of the fMRI cue-based task (two runs), the fMRI picture-based task (single run), and a T1-weighted structural scan. The order of the picture-based and cue-based tasks was randomized across participants, and the T1 scan was always conducted between the first and second runs of the cue-based task to avoid having a continuous period of electrical shocks. The whole MRI acquisition session lasted around 1 hour. The participant was not required to make a response (such as a rating) in the scanner to maximize number of trials and statistical power.

The cue task was a 2 X 2 X 3 within-subject event design (self/other pain X hand/foot pain X no/low/high pain levels). There are 12 conditions in total, and each condition is repeated 15 times giving 180 trials per run plus an additional 10 blank trials (no visual and no pain stimuli). At the start and end of the block, a 12 second blank screen was shown. Trials were presented in a pseudo-random order such that the same condition never appeared twice in a row. We aimed to include as many repetitions of each trial type as possible to increase the signal–noise ratio, SNR, for single trial estimation. The trial sequence and blank trial placement were optimized for stimulus onset asynchrony and robust HRF estimation. Participants viewed a black screen that was divided into two halves labelled “self-pain” (i.e., the participant) and “other pain” (i.e., the experimenter). In one run, “self-pain” was on the left and, in the other run, it was on the right side of the screen. Pre-recorded (4 s) videos of the experimenter’s hand or foot with electrodes attached were shown in the “other pain” section of the screen. Participants were told that the videos were a live-feed of the experimenter. The same videos were used in all conditions such that they were uninformative about the level or subject of pain. This ensures that the self-pain (physical) and other pain (vicarious) conditions are visually identical to each other when averaged across trials and runs. The trial sequence is shown inFigure 1.

For a given trial, in the first 1 s, an arrow indicates which person will receive the shock, and in the next second, a shock is delivered (in the self-pain condition) and, simultaneously, a visual cue indicates the intensity of the shock: either 1, 2, or 3 lightning bolts are displayed which correspond, respectively, to values of 0 (no pain), 5 (low pain), and 10 (high pain) on the subjective scale (noting that the physical value can differ between hand and foot). Cues were always valid: a hand cue never leads to foot stimulation, the “other” cue never leads to “self” stimulation, and so on. An inter-trial interval (ITI) of 2 s was used throughout the task.

At the end of the session, participants answered four questions about their experience of the cue-based task. (1) How unpleasant was it when the shock was on you? (0 = strongly pleasant, 10 = strongly unpleasant). (2) How unpleasant was it when the shock was on the researcher? (0 = strongly pleasant, 10 = strongly unpleasant). (3) Did you think the electrical stimulation delivered to the researcher was real? (0 = strongly disagree, 10 = strongly agree). (4) When the other person got the electrical stimulation, did you tend to (0—think about yourself—10 think about the other person).

#### 2.2.4 MRI data acquisition

A 3T Siemens Prisma scanner with a 64-channel head coil was used to acquire all images. Functional images were acquired using the Human Connectome Project (HCP) gradient echo EPI sequence, with a multiband acceleration factor of 8; TR = 0.8 s; TE = 33.1 ms; 52 degree flip angle; FOV = 208 x 180 mm; 72 slices with slice thickness of 2 mm and isotropic 2 mm voxels. Two SpinEcho Field maps with reversed phase-encode blips in Anterior to Posterior and Posterior to Anterior were acquired with the same parameters as the functional images. A high-resolution structural T1-weighted image was acquired with 3D MPRAGE sequence (TR = 2.4 s; TE = 2.14 s; 8 degree flip angle; FOV = 224 x 224 mm and 0.8 mm isotropic voxels).

### 2.3 Neuroimaging analyses

#### 2.3.1 fMRI pre-processing

Pre-processing was performed using a pipeline combining tools from the SPM12 suite (Wellcome Department of Imaging Neuroscience, London, UK), FSL (D. T. Smith et al., 2004), and Freesurfer (Fischl et al., 2002).

*Anatomical preprocessing.*T1-weighted anatomical volumes were corrected for gradient non-linearities based on scanner calibration measurements. Non-brain data were removed from each T1 volume using the FMRIB Brain Extraction Tool (S. M. Smith, 2002), then aligned to the Montreal Neurological Institute (MNI) 152 standard template anatomical image using FSL’s FMRIB’s Linear Image Registration Tool with 12 degrees of freedom (Jenkinson et al., 2002). Each volume was inspected for image artefacts and tissue contrast and rejected if deemed of poor quality. Cortical surface representations were generated from the T1 volume (1 mm resolution) using FreeSurfer version 6 beta (build-stamp 20161007) with the -hires option. Mappings to fsaverage space were calculated and various standard atlases were mapped for each subject. Final surface visualizations were consolidated into movies, to be visually inspected for errors in surface generation. When cortical reconstructions showed inaccuracies, manual adjustments of the grey/white matter contrast were made and Freesurfer segmentation and surface construction re-run to ensure high-quality surface generation for all subjects.

*Functional preprocessing.*Both temporal and spatial preprocessing were performed on the fMRI data. Field maps were estimated and applied using FSL’s top-up utility, to correct for image distortions (Andersson et al., 2001). Cubic interpolation was performed on each voxel’s time series data to correct for differences in slice acquisition times and to obtain an integer sampling rate of 1.0 s. Rigid-body motion parameters were estimated from the undistorted EPI volumes using the SPM12 utility spm_realign. Finally, cubic interpolation was performed on each slice time-corrected volume to compensate for the combined effects of EPI distortion and motion. No spatial smoothing or temporal filtering was performed. The average of the preprocessed functional volumes was co-registered to the T1 anatomical volume (affine transformation estimated using a 3D ellipsoid that focuses the cost metric on cortical tissue). This resulted in a transformation that maps the EPI data to the participant native brain anatomy volume. Functional data were then projected onto the freesurfer-generated cortical surface, all subsequent functional preprocessing and analysis were performed in this space.

Data scrubbing was also implemented to address head motion issues. No participant was excluded from further analyses because of excessive head motion. To compare the effects of head motion amongst different groups, we calculated framewise displacement (FD) head motion for each task. This calculates the relative head motion of each time point to its prior time point (Power et al., 2014). One-way ANOVA was performed on FD amongst the three groups. Results showed that there was no significant difference amongst the three groups in either run (run 1: F(2,63) = 0.818, p = 0.446; run 2: F(2,63) = 0.446, p = 0.259).

#### 2.3.2 First level single-subject fMRI analyses

For the univariate analyses, a 4 mm FWHM smoothing kernel was applied to the functional images for the GLM analyses. No smoothing was applied to the data used in the multivariate analysis.

The 1 s shock and 2 s cue presentation were modelled using regressors that convolved with the canonical hemodynamic response function. The blank trials that remained served as an implicit baseline. GLM-denoise was applied (available MATLAB code athttp://kendrickkay.net/GLMdenoise/) which derives the optimal number of principle components to use as noise regressors (Kay et al., 2013). Further data analysis was conducted using custom scripts in MATLAB (version 2019b; The MathWorks, Inc., Natick, MA, USA). This has been run twice, once in a simple way that generates one beta value per voxel (typical for univariate approaches), and a second version that splits the different trials into three different beta estimates per voxel (for the multivariate approaches). This ensured that there were separate train and test data available for the classifier. Specifically, the 15 repetitions (averaged across runs) are split into three: the first is made from instance 1,4,7,10,13, the second is made from 2,5,8,11,14, and the third from 3,6,9,12,15.

#### 2.3.3 Univariate general linear model (GLM) analyses

A second-level GLM was run on the data, assessing the self versus other contrast within groups and also directly comparing the difference contrasts between groups.

Given that pain had three levels, these were modelled as continuous variables in a Pearson’s correlation and using three separate beta values per condition (smoothed). This was done separately for self-pain and other pain conditions. Thus, each correlation was derived from 18 values (3 pain levels x 2 body parts x 3 beta values) generating an r-map for each participant. These were Fisher transformed (r-to-z) for between-group statistical comparisons.

Whole brain cluster-level inferences were made using Threshold Free Cluster Enhancement (TFCE) (S. M. Smith & Nichols, 2009), which avoids the use of an a priori cluster-forming threshold. The CoSMoMVPA tool-box (Oosterhof et al., 2016) was used to implement TFCE for surface data. TFCE scores are weighted based on the spatial extent of each cluster, and the cluster height. For each contrast (as specified above), permutation tests of 1000 permutations were used to derive a null distribution. The observed effects were then compared with this null distribution. All TFCE-corrected maps show effect size measures with an FWE-corrected threshold of p < 0.05 (2-tailed). TFCE has the advantage of being associated with a lower false-positive rate than traditional cluster-size tests based on random field theory (Salimi-Khorshidi et al., 2011).

#### 2.3.4 Multi-voxel pattern analysis (MVPA)

Regions of interest (ROI) within the pain matrix were identified based on the HCP (Human Connectome Project) multimodal anatomical atlas (Glasser et al., 2016) together with the results of the univariate analysis (main contrast of self-pain > no pain). The regions of interest were the insular cortex, mid-cingulate cortex, somatosensory cortices, and all three of these regions—considering left and right hemispheres separately (i.e., 8 ROIs in total). By using different ROIs, including across hemispheres, the aim was to make the results more interpretable (understanding how different regions contribute more or less) and by putting them together, we gain an estimate of overall prediction accuracy. The insula ROI comprised voxels in the following regions of the HCP atlas: PoI2, MI, AVI, AAIC, PoI1, FOP4, FOP3, FOP2, FOP5, Ig, and PI. The cingulate ROI comprised voxels in the following regions of the HCP atlas: 24dd, 24dv, p24pr, 33pr, a24pr, and p32pr. The somatosensory ROI comprised voxels in the following regions of the HCP atlas: BA3B, BA3A, BAOP4, BAOP1, BAOP2-3, BA1, and BA2. Within each ROI, voxels with near zero variation, that is, poor predictive ability, were removed (using the “cosmo_remove_useless_data” function).

The split beta values (three per condition) from the first-level analyses for each participant were used as the input for MVPA. All analyses in this stage were performed using CoSMoMVPA (Oosterhof et al., 2016) in Matlab using LDA (linear discriminant analysis). Three different classifiers were developed. One classifier was trained to predict pain (high/low vs. none) from the self-pain trials, and one classifier was trained to predict pain (high/low vs. none) from the other pain trials. These classifiers were then used in cross-classification to determine the extent to which self-pain can predict other pain (and vice versa)—that is, to address the question of shared representations in a multivariate manner. A third classifier was trained to distinguish self-pain (high, low, none) from other pain (high, low, none).

For the pain classifiers, for each participant, there are 18 sets of data (2 body parts X 3 pain levels X 3 beta estimates) which were divided into test and train sets using 6-fold cross-validation. The classifier was trained to distinguish presence of pain (high or low) from its absence. An imbalanced classifier (12 pain example, 6 no pain examples) can trivially achieve above chance (>0.5) performance (Basha et al., 2022), so a balancing procedure in Matlab was added (cosmo_balance_partitions). This reduced the test data to 12 sets drawn equally from both categories.

For the self–other classifier, for each participant, there were 36 sets of data balanced between conditions (18 self, 18 other) which were divided into test and train sets using 6-fold cross-validation.

## 3 Results

### 3.1 Pre-fMRI pain thresholds and empathy for pain behavioural ratings

Recall that the aim of the pre-test session was to establish individual pain stimulation levels to carry to the fMRI session, and also to introduce participants to the empathy for pain task (e.g., to make it plausible that both people receive shocks). The results are shown inFigure 2.

**Fig. 2.**
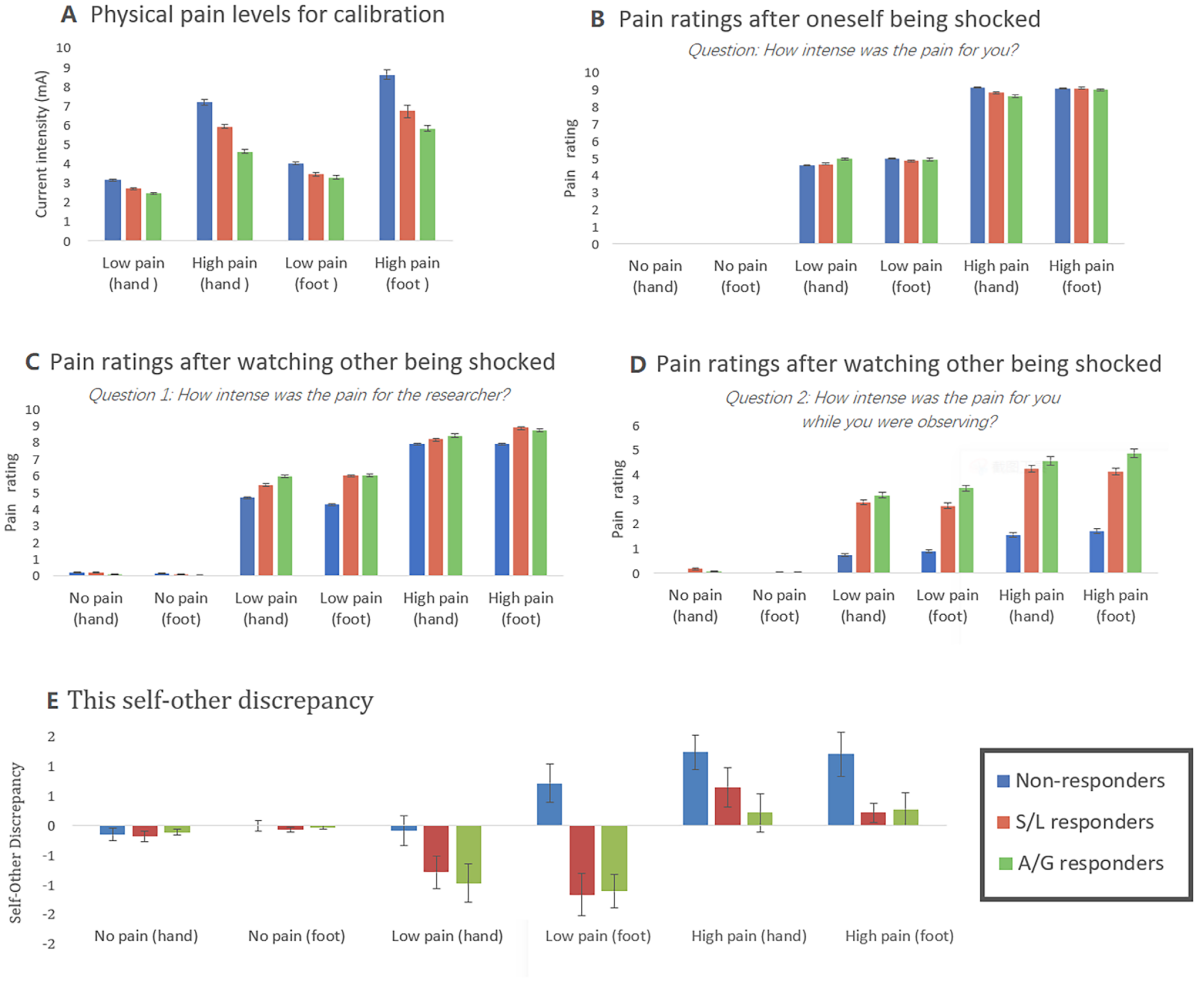
Behavioural results (before neuroimaging session). (A) The averaged pain thresholds for low-pain and high-pain conditions. (B) The averaged pain ratings after the subject has been shocked. (C) The average pain ratings for imagining how much pain the other person experienced. (D) The average pain ratings for vicarious pain felt by the observer when watching the experimenter being shocked. (E) The average difference values of pain ratings between self-pain (B) and other pain (C), non-responders tend to rate their own pain as worse than that of other people, but this tendency is reduced/reversed in responders. Error bars show ± 1 SEM.

The levels of electrical current (mA) subjectively rated as 5/10 (low pain) and 10/10 (high but tolerable) were analyzed as a 2 x 2 x 3 mixed ANOVA contrasting pain level, body part, and group. There was no main effect of group (F(2,62) = 2.600, p = 0.082, η^2^= 0.077) or group X body part interaction (F(2,62) = 0.112, p = 0.894, η^2^= 0.004), but there was a pain level X group interaction (F(2,62) = 3.396, p = 0.040, η^2^= 0.099). That is, the vicarious pain responders are less tolerant of physical pain at higher intensities (notably the AG group). Post hoc comparisons, collapsing across body part and pain level, revealed significant differences between AG and non-responders (t(43) = 2.259, p = 0.029) with SL being intermediate between other groups.

These calibrated levels were carried forward to the behavioural empathy for pain task. Cues depicting one, two, or three lightning bolts predicted pain level (one lightning bolt was no pain, and the others were the low and high levels). The cue preceded an electric shock being delivered to the participant or experimenter’s hand or foot, which were then rated on a 0–10 scale (the dependent variable). In the self-pain condition, a 3 x 2 x 3 mixed ANOVA contrasting pain level, body part, and group revealed no main effect of group (F(2,62) = 0.112, p = 0.894, η^2^= 0.004) and no interactions with group (pain level X group F(4,124) = 0.756, p = 0.894, η^2^= 0.004; body part X group F(2,62) = 0.034, p = 0.967, η^2^= 0.001). This stands in contrast to the ratings of pain when the other person was stimulated where group differences were found. Here, participants were asked two questions: how much pain they believed the other person felt and how much pain they felt themselves (i.e., on their own body)—the latter being vicarious pain. Here, a significant group effect was found on both measures: other pain F(2,62) = 4.419, p = 0.016, η^2^= 0.125; vicarious pain F(2,62) = 9.428, p < 0.001, η^2^= 0.233. In both cases, there was a significant pain level X group interaction, showing that the difference between groups emerged at higher pain levels (other pain F(4,124) = 5.034, p = 0.001, η^2^= 0.140; vicarious pain F(4,124) = 7.894, p < 0.001, η^2^= 0.203). For the other pain question, there was also a significant body part X group interaction such that vicarious pain responders attributed higher pain to feet (F(2,62) = 5.260, p = 0.008, η^2^= 0.145).

Whereas non-responders tend to rate their own pain as worse than that of other people, this tendency is reduced/reversed in responders. This self–other discrepancy is depicted inFigure 2E(calculated as the difference scores betweenFig. 2B and C) and, in itself, shows a significant group difference (F(2,62) = 6.587, p = 0.003, η^2^= 0.175) and a group X pain-level interaction (F(4,124) = 5.370, p = 0.001, η^2^= 0.148).

### 3.2 Post-fMRI behavioural ratings

Following the fMRI, participants tended to believe that the experimenter did really receive pain (on a 0–10 scale; non-responder = 6.240, SD = 2.260; SL = 7.100, SD = 2.222, AG = 7.250, SD = 2.511) and this did not differ across the groups (F(2,64) = 1.261, p = 0.290). Similarly, participants reported that their own pain was unpleasant (non-responder = 7.200, SD = 1.658; SL = 6.750, SD = 1.164, AG = 6.450, SD = 1.572) and believed that the other person found it unpleasant (non-responder = 5.080, SD = 1.320; SL = 5.600, SD = 1.535, AG = 5.350, SD = 1.755), and these ratings also did not differ across groups (F(2,64) = 1.439, p = 0.245 and F(2,64) = 0.646, p = 0.528, respectively). However, the groups differed significantly on their self versus other orientation on the vicarious pain trials (F(2,64) = 5.474, p = 0.006). That is, non-responders reported a self-focus (<5 on the scale) whilst the other person was receiving pain (mean = 4.280, SD = 2.189), the two responder groups reported a greater other focus (S/L = 6.050, SD = 1.877, A/G = 6.050, SD = 2.188).

### 3.3 Univariate analyses of self versus other

**Fig. 3.**
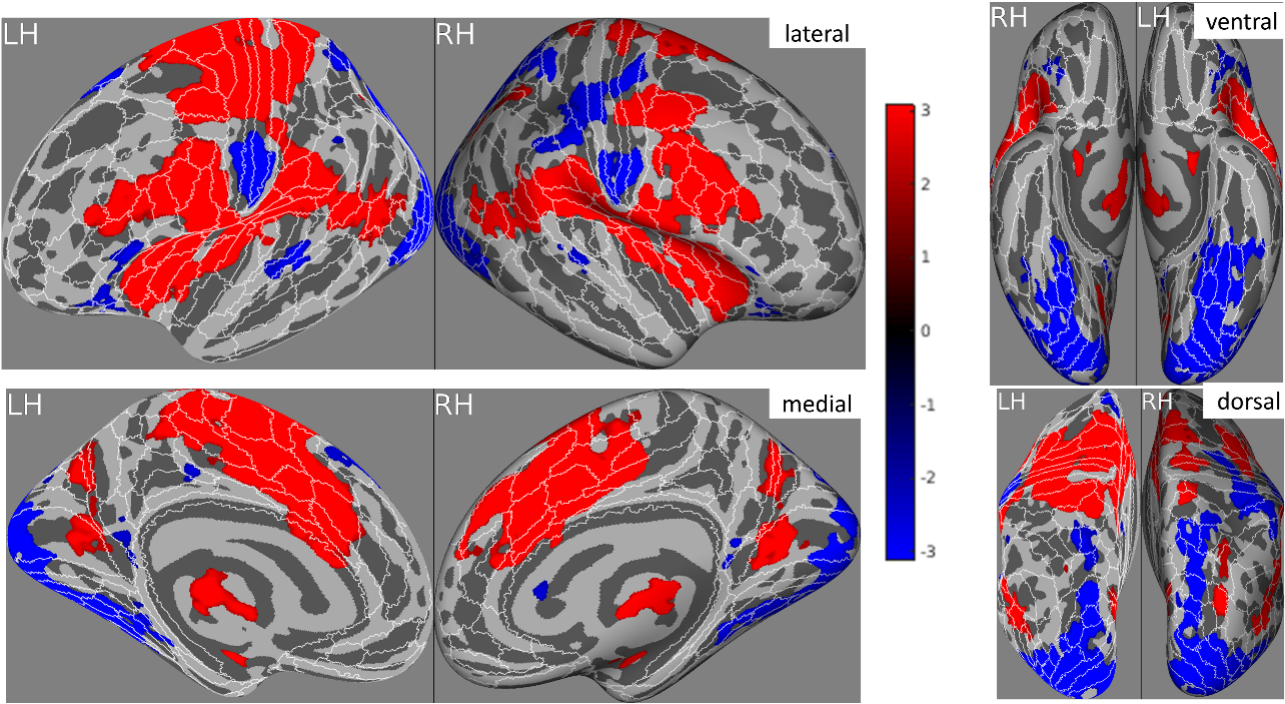
The brain activation of all self-conditions versus all other conditions after correction for multiple comparisons across all participants (LH: left hemisphere; RH: right hemisphere). Maps display the t-statistic effect size measure, and constrained using Threshold Free Cluster Enhancement. White outlines and labels overlaid on the brain depict the boundaries of the HCP MMP anatomical atlas. All data are displayed on the inflated fsaverage cortical surface.

The contrast of self > other revealed significant activation in the somatosensory cortex (more strongly on left, noting that the right side of body was stimulated), premotor cortex, frontal eye field, inferior frontal gyrus, temporoparietal junction, posterior parietal cortex, insula, frontal operculum, inferior parietal cortex, supplementary and cingulate eye field (SCEF), and mid-cingulate cortex (Fig. 3). There are also some negative activations (i.e., other > self) in somatosensory cortex (inferior region, likely corresponding to face area) and visual cortex. There were no significant group differences, although these were expected. Specifically, greater pain-related activity for “other pain” was predicted to manifest itself as a smaller self–other difference in vicarious pain responders.

### 3.4 Univariate analyses of pain intensity

The previous section compared self versus other conditions (collapsing across levels of pain intensity). Here different levels of pain intensity are compared, separately for self and other. Specifically, we correlate the different levels of pain (as the subjective values 10, 5, 0) with extracted voxel-wise activity (beta values).

For the self condition, this reveals widespread activity largely consistent with that reported above (self–other) and the literature on the “pain matrix”—seeFigure 4. No group differences amongst the r-maps are found, noting that we did not expect a priori any group differences in physical/self-pain.

**Fig. 4.**
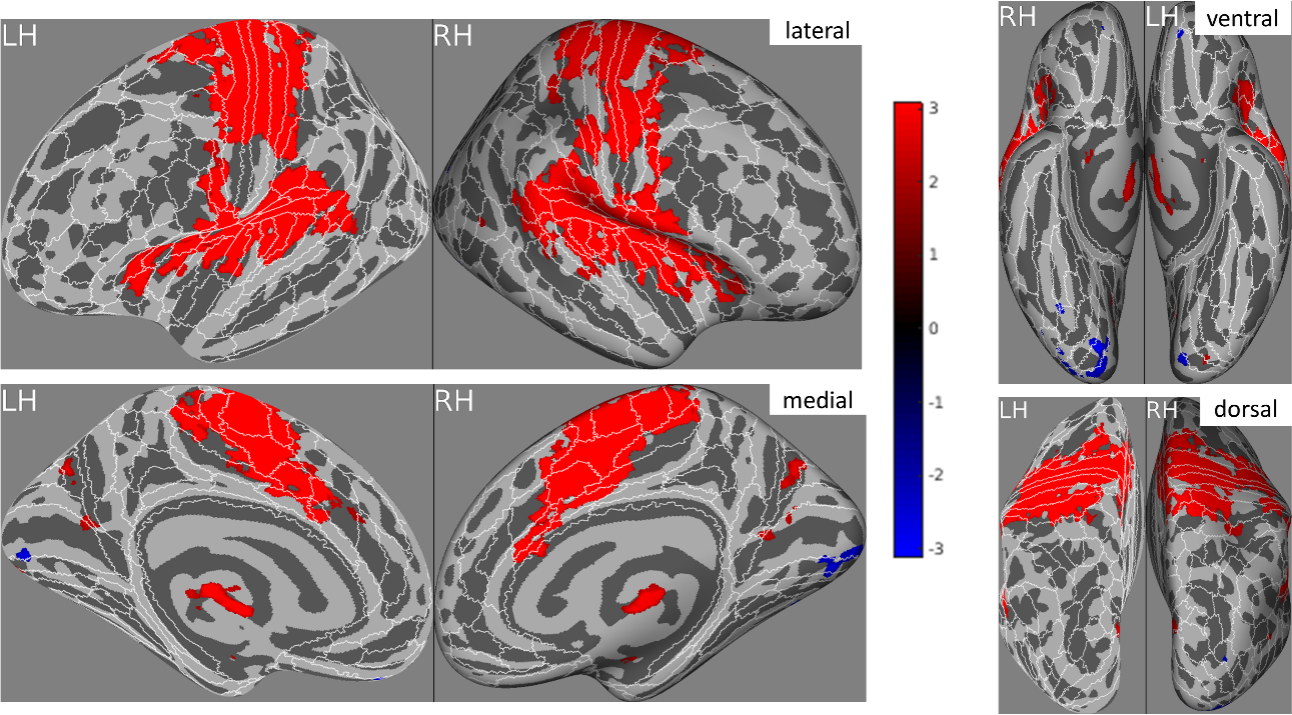
The correlations between self-pain brain activities and three pain levels (no, low, high) after correction for multiple comparisons. The effect size measure being displayed is the group averaged r statistic (after Fischer transformation and inverse Fischer transformation), and constrained using Threshold Free Cluster Enhancement. White outlines and labels overlaid on the brain depict the boundaries of the HCP MMP anatomical atlas. All data are displayed on the inflated fsaverage cortical surface.

For the other condition, there were no significant differences (main effect) beyond early visual cortices, and no significant differences between groups. That is, the univariate analyses of vicarious pain do not resemble those of physical pain. However, a stricter test of “shared representations” comes from our multivariate analyses below.

### 3.5 Multivariate analysis (MVPA)

Recall that there were three classifiers trained to predict: self-pain versus self no pain, other pain versus other no pain, and self versus other (across all pain levels) using ROI data from regions of the pain matrix. Note also that we can additionally perform cross-classification such that a classifier trained to predict self-pain can be tested on other pain (and vice versa). Overall performance of the classifier is assessed relative to chance (accuracy of 0.5, using one-sample t-tests) and considering differences in classification accuracy by region and group using mixed ANOVA (3 groups X 4 ROIs X 2 hemispheres). Significant levels of cross-classification (irrespective of group) would be indicative of shared neural substrates between physical and vicarious pain. Beyond that, individual differences relating to the other pain classifier or the self–other classification are predicted as main effects of group and/or a group X region interaction. (Note there is no reason to expect group differences relating to classification of physical pain in the self condition).

The performance of the three classifiers, and the two additional cross-classification results, is shown inFigure 5. Performance on the self–other classifier was high and all regions were able to discriminate above chance (all p’s < 0.001). There was a significant main effect of group (F(2,61) = 4.099, p = 0.021, η^2^= 0.118) such that non-responders had higher classification accuracy than the responder groups, the latter being consistent with self–other confusion. Group status did not interact with region or hemisphere (group X ROI: F(6,183) = 1.251, p = 0.283, η^2^= 0.039; group X hemisphere: F(2,61) = 2.081, p = 0.134, η^2^= 0.064; group X ROI X hemisphere: F(6,183) = 1.689, p = 0.126, η^2^= 0.052). That is, group differences are robust across all the regions considered. In addition, there were main effects of hemisphere (F(1,61) = 5.286, p = 0.025, η^2^= 0.080) and ROI (F(3,180) = 14.254, p < 0.001, η^2^= 0.189). The whole pain matrix was a better classifier than individual regions, and the left hemisphere had generally better classification accuracy (noting that pain was delivered to the right side of the body).

**Fig. 5.**
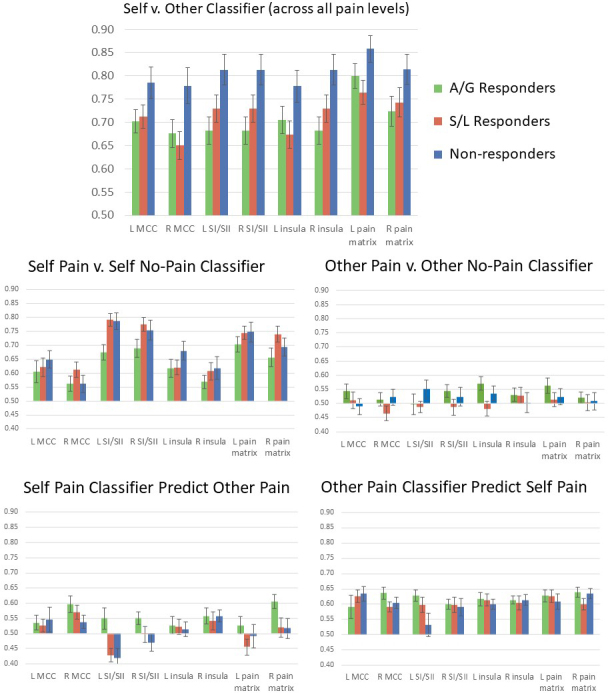
Classifier performance (mean ± 1 SEM, chance = 0.5) divided by group for classifiers trained, within each individual, to predict self versus other (top), self-pain versus self no pain (middle left), and other pain versus other no pain (middle right). The lower panels show cross-classification performance using self-pain to predict other pain (bottom left) and other pain to predict self-pain (bottom right).

For the self/physical pain versus no pain classifier, performance overall was high (with each of the eight regions classifying above chance, all p’s < 0.001). There was no main effect of group (F(2,60) = 2.429, p = -0.097, η^2^= 0.075), but significant main effects of hemisphere (F(1,61) = 6.846, p = 0.011, η^2^= 0.102) and ROI (F(3,180) = 46.378, p < 0.001, η^2^= 0.436). The best discriminating region was left somatosensory cortex. There were no significant interactions. For the other/vicarious pain versus no pain classifier, performance overall was at chance in all eight regions (p > 0.05) and, hence, was not analyzed further.

There was some evidence for cross-classification in both directions. The self-pain classifier could predict (above chance > 0.5) other pain/no pain in three regions: left MCC (p = 0.035), right MCC (p < 0.001), and right insula (p < 0.001). There were significant main effects of hemisphere (right > left; F(1,61) = 16.914, p < 0.001, η^2^= 0.220) and ROI (F(3,180) = 4.277, p = 0.006, η^2^= 0.067), and an ROI X hemisphere interaction (F(6,183) = 3.855, p = 0.010, η^2^= 0.139). There was a group X hemisphere interaction (F(6,183) = 4.861, p = 0.011, η^2^= 0.052), which was driven by good cross-classification accuracy (self-pain predicting other pain) from one of the responder groups (AG) in right hemisphere regions. For the opposite cross-classification—other pain predicting self-pain—all regions performed above chance level of 0.5 (p < 0.001), and the ANOVA produced one significant finding, namely a triple interaction between group X region X hemisphere (F(6,180) = 2.443, p = 0.027, η^2^= 0.075). Post hoc examination of group differences (one way ANOVA) across all eight ROIs revealed a significant effect in left somatosensory cortex (F(2,61) = 3.795, p = 0.028), such that responders have higher cross-classification accuracy than non-responders in that region. In other words, a classifier trained to distinguish other pain/other no pain is better able to distinguish self-pain/self no pain in people with vicarious pain experiences in left somatosensory cortex.

## 4 Discussion

There is ongoing debate about whether vicarious pain (“other”) and physical pain (“self”) share the same neural resources and, in this study, we take a novel individual differences approach to this question based on previous observations that some people report consciously experiencing the pain of others (vicarious pain responders) but most people do not. We predicted that neural substrates involved in physical pain would be more activated by vicarious pain (here, cues depicting strength of electric shock to an observed individual) in responders. Further, that in multivariate analyses, the pattern of responses for physical and vicarious pain would be more predictive of each other in vicarious responders. Although our univariate analyses did not point to group differences, we see various differences using MVPA and in behavioural responses that suggest a greater similarity between self and other measures of pain in vicarious responders relative to non-responders. Overall, our two groups of vicarious pain responders (S/L and A/G) were similar to each other, although we hypothesized that the S/L group (who report localized pain experiences with sensory qualities) may differ from the A/G group in primary somatosensory cortex (in either univariate or multivariate analyses). These findings are discussed in detail below and linked to theoretical accounts of the neural bases of vicarious pain perception.

### 4.1 Behavioural data: Vicarious pain responders are more sensitive to both self and other’s pain

The pain calibration in the pre-test session found that responders have a lower pain threshold than non-responders—that is, they are more sensitive to physical pain. This parallels the observation that these same participants are, by definition, more sensitive to vicarious pain. (Note that for neuroimaging, the groups were matched in terms of individual subjective pain levels, so we do not observe group differences in the physical pain conditions.) This is in line with previous work that explores the relationships between pain sensitivity and vicarious pain (Ren et al., 2020). For example, studies have found that when there is a lack of emotional cues, patients with congenital insensitivity to pain will greatly underestimate the pain of others, and at the same time, the activation of brain areas related to empathy is also reduced when looking at the pain of others, compared with the control group (Danziger et al., 2006,^2009^). Amongst healthy participants, the ERP signals related to vicarious pain correlate with each participant’s pain sensitivity to electrical stimulation (Liu et al., 2019). Some animal studies also support the result showing modulation of firsthand pain sensitivity in rats when exposed to cage mates experiencing pain (Langford et al., 2006).

Moreover, during pre-test, we also found responders rate the pain of others differently. Here, we asked two questions. One relates to vicarious pain itself—the degree to which you, as an observer, experience pain. Consistent with our initial groupings, the non-responders give near-zero ratings but vicarious pain responders report pain. This suggests that the cueing paradigm is effective (despite not showing overt injury). An additional question was to rate how much pain they thought the experimenter experienced, that is, a condition in which their own vicarious response is not directly relevant. Here we find that non-responders tend to rate their own pain as worse than that of others (even when the cue indicating no pain, low pain, or high pain is the same) but responders show a more equal response between self and other.Coll et al. (2017)argue that measures of empathy should clearly delineate between affect sharing and emotion identification which are putatively independent mechanisms. However, our results speak against the suggestion that they are completely separate. People who share the pain of others also tend to infer higher levels of pain in others. The extent to which people engage in different kinds of emotion regulation strategies (e.g., to focus away from the distress of others) may determine the extent to which these processes are separate or interconnected. Similar effects are reported byColl et al. (2017)in a re-analysis of the placebo analgesia study ofRuetgen et al. (2015). This study also asked separately about inferred pain in others (“How painful was this stimulus for the other person?”—emotion identification) and the unpleasantness of one’s own responses (affect sharing). Using mediation analyses, they showed how these judgements are related, with affect sharing being mediated by emotion identification. Whilst our study did not include a measure of unpleasantness per se, it is comparable in the sense of including measures of pain sharing (in oneself) and pain identification (in others).

After the scanning, we also found non-responders reported a stronger self-focus whilst the other person was receiving pain, whilst the two responder groups reported a greater other focus. It may be possible to flexibly shift between a self-focus (in which other perspectives are inhibited) and a focus on others (inhibiting one’s own beliefs and feelings). But it may also be the case that certain groups or individuals have a default style towards one or the other (Bird & Viding, 2014) and our data suggest that this applies to vicarious pain responders. That is, reduced self-focus may be the route to greater affect sharing, including vicarious pain. A final possibility is that there could be greater shared focus in vicarious pain responders, which is sometimes postulated to occur in particular contexts such as joint action (Bieńkiewicz et al., 2021).

### 4.2 Neuroimaging data: Responders show less self–other discrimination in MVPA

For physical pain, our study demonstrates that neural activity in the pain matrix including somatosensory cortex, MCC, and insula was positively correlated with the intensity of electrical stimulation. Comparable results were not found when correlating the intensity of implied pain delivered to the other person (in vicarious pain conditions). Univariate activity in somatosensory cortex to vicarious pain is not consistently found in cue-based paradigms (Lamm et al., 2011). We did not find any group differences in the vicarious pain univariate analyses, in contrast to our predictions and prior research (Osborn & Derbyshire, 2010;Grice-Jackson et al., 2017b). These discrepancies are hard to interpret given differences in paradigms relating to both stimuli (images of pain) and task (ratings vs. passive viewing). We do not make strong claims about the null group results in univariate analyses, particularly in light of the group differences that do emerge with multivariate (MVPA) analyses which were carried out in the same pain-related regions identified in the univariate analyses (for further discussion of reasons for discrepancy between these approaches seeDavis et al., 2014).

Two kinds of classifiers were developed. One that distinguished pain (high/low) against no pain, and one that distinguished self from other (across all pain levels). For the pain-based classifiers, we were also able to employ cross-classification such that a classifier trained to predict self-pain (from self no pain) can then be tested to make predictions about the vicarious pain trials, and vice versa. The most robust MVPA findings—statistically reliable across all regions of the pain matrix—was that there was higher self versus other classification accuracy in non-responders than the two responder groups. Or, in other words, neural signatures of physical pain and vicarious pain are less discriminable in people who report feeling the pain of others. These results are consistent with the behavioural ratings, summarized above, in which responders tend to give more similar ratings for self-pain and other pain. Both neuroimaging and behavioural results suggest a style of processing in which self and other are less sharply delineated. It is also consistent with a second published study (on the same participants) that used a picture-based task in which images of hands and feet in pain were contrasted with no pain images (Li et al., 2024). A whole-brain fMRI biomarker developed to predict physical pain could better discriminate BOLD responses to these images in responders than non-responders.

For the pain versus no pain classifiers, we find equivalent levels of performance across the groups. But group differences emerge when they are used in cross-classification (vicarious pain predicting physical pain, and vice versa). Cross-classification between vicarious and physical pain has been reported in some studies (Corradi-Dell’Acqua et al., 2016;Zhou et al., 2020) but not in others (Krishnan et al., 2016). Here we show that the extent to which cross-classification is successful is modulated by individual differences (shown by group X region interactions for both types of cross-classification). In regions such as left somatosensory cortex, cross-classification is more accurate in responders than in non-responders; that is, vicarious pain can better predict self-pain. The fact that cross-classification between vicarious pain and self-pain outperforms classification within vicarious pain itself (classifying other pain from other no pain) could reflect the fact that the classifier is trained on weakly discriminating information (resulting in performance that is numerically but not significantly above chance) but tested, during cross-classification, on strongly discriminating information (resulting in more statistically robust differences).

Thus, the results from the MVPA are coherent. Responders have more similarity in the neural resources of physical pain and vicarious pain such that one can predict the other (in cross-classification) but also such that they are more confusable when pitted together in self–other classification.

### 4.3 Wider implications and limitations

The present research offers further support for the idea that pain-like experiences can arise in the absence of injury, consistent with contemporary definitions of pain as “An unpleasant sensory and emotional experience associated with, or resembling that associated with, actual or potential tissue damage” (Raja et al., 2020). It differs from some previous attempts to infer pain, in a purely third person way, from neuroimaging data in situations such as social rejection (Eisenberger, 2015) because, in the present study, at least some participants are reporting first-person experiences of pain. That is, we are not directly inferring pain from neuroimaging but, instead, we are using neuroimaging to corroborate self-reported experiences.

Theories of empathy typically divide it into multiple component mechanisms of which affect sharing is only one (Decety & Svetlova, 2012;Zaki & Ochsner, 2012). Vicarious pain would be one particular example of affect sharing, although noting that pain has a shared sensory component too. Our ROI results support the conclusion that vicarious pain involves both sensory (e.g., somatosensory cortices) and affective (e.g., insula) brain regions. Additional mechanisms have been variously labelled as self–other control or perspective taking, but with the common assumption that these mechanisms regulate the extent to which affect sharing is enhanced or suppressed. Some accounts argue that suppressing affect sharing is functionally adaptive because it enables a compassionate response that is not motivated by alleviating one’s own personal distress (Singer & Klimecki, 2014). Translating these theories to vicarious pain, one could assume that there is either an enhancement of shared representations per se (hyperactive mirroring) or individual differences in the regulation/suppression of shared representations (Ward & Banissy, 2015). The present study does not unequivocally adjudicate between these accounts, although the behavioural ratings point to group differences in perspective taking (non-responders are more self-focused which may protect against vicarious pain). Individual differences could occur in terms of both their default orientation (towards self or other) and degree of flexibility in that orientation (some people may lack an ability to shift) (Bird & Viding, 2014). We speculate that vicarious pain responders have a default style that is biased towards affect sharing (focus on other) and less flexible.

One limitation of the present research is that it does not speak of the “bigger picture” of vicarious pain responders in terms of either candidate neural mechanisms (outside of the pain matrix) or the wider behavioural phenotype (beyond vicarious pain itself). The right temporo-parietal junction has been shown to have structural differences (less grey matter;Grice-Jackson et al., 2017a) and functional connectivity differences (Grice-Jackson et al., 2017b) in vicarious pain responders, but the region was not explored here as we focused on shared representations (the region did not generate group differences in the whole-brain univariate analysis). The data are shared for further analyses. This region is important because it is implicated in self–other control and perspective taking (Bzdok et al., 2013), including in tasks involving mentalizing (which typically involve suppressing egocentric thinking). With regard to behaviour,Zaki et al. (2016)note the importance of documenting the psychological “tuning curve” for vicarious pain. Thus, although we can identify sub-groups of people within the general population who reliably report vicarious pain, this does not mean that the differences are restricted only to pain. In previous research, we have found that people defined as “vicarious pain responders” are more likely to report vicarious touch (Ward et al., 2018) and emotion contagion (Botan, Bowling, et al., 2018). Finally, although we have documented two different types of vicarious pain (S/L and A/G), we do not find reliable differences between them in the present study. This may either reflect low statistical power or an absence of true differences (at least on the measures used here). However, they have been shown to differ on measures such as rubber hand illusion (Botan, Fan, et al., 2018) and interoception (Botan et al., 2021;Bowling et al., 2019).

Other limitations are related to this particular choice of experimental design and the potential presence of other uncontrolled variables that could either confound or add noise.Gamble et al. (2024)discuss the potential impact of “external factors” such as situational factors and observer–target relationship on affect sharing. In our study, these factors would include the believability of the paradigm and implicit and explicit attitudes towards the experimenter (including those relating to gender and race). Although there is no a priori reason to assume these differ according to group (responder vs. non-responder), we have very little evidence either way (beyond the end-of-scan ratings). Even if the groups are matched on average to external factors, there is likely to be variability both between individuals and within individuals (e.g., the believability may change over time) that adds noise to the results. Gender differences would be another potential source of inter-individual variability that we did not model (Christov-Moore et al., 2014). The evidence for women being more empathic than men is far stronger for self-report measures such as questionnaires than for neuroscientific markers of empathy, such as from EEG (Pang et al., 2023). Women are more likely to be classed as vicarious pain responders (Ward & Li, 2022). The present results cannot be trivially dismissed due to gender (we matched for it across groups), but the question of whether gender and responder status interact in some way is unknown.

In summary, we find evidence consistent with shared representations between vicarious pain and physical pain from both behavioural measures and multivariate analyses. Future research needs to determine the conditions in which shared representations are more or less likely to be recruited, including individual differences.

## Data Availability

This is available under CCO license viahttps://osf.io/63p2g/.
